# Design, Synthesis, Anti-Tumor Activity and Molecular Docking Studies of Novel Triphenylphosphine-Containing Formononetin Derivatives

**DOI:** 10.3390/ijms26115280

**Published:** 2025-05-30

**Authors:** Hongjuan Cui, Yan Zhao, Wei Li, Huanjie Cui, Jiahong Han, Enbo Cai

**Affiliations:** College of Chinese Medicinal Material, Jilin Agricultural University, 2888 Xincheng Street, Changchun 130118, China; 19969597086@163.com (H.C.); zhaoyan@jlau.edu.cn (Y.Z.); liwei7727@126.com (W.L.); 13179257902@163.com (H.C.)

**Keywords:** formononetin, mitochondrial, targeted drug delivery, triphenylphosphine, molecular docking, *SHMT2*

## Abstract

Formononetin is widely used in anti-tumor research, but its poor water solubility leads to low absorption and poor utilization efficiency in vivo, limiting further development. The triphenylphosphine cation was partially attached to the 7-position hydroxyl group of formononetin to specifically target it into the mitochondria of tumor cells to enhance the anti-tumor effect. Detailed structural characterization via ^1^H-NMR and ^13^C-NMR analysis confirmed the physical properties and chemical structures of 21 newly synthesized derivatives. The effects of these derivatives on tumor cells were assessed by in vitro and computational methods. MTT results from four tumor cell lines showed that formononetin derivatives containing triphenylphosphine had stronger anti-tumor activity than formononetin and exhibited more cytotoxic effects in cancer cells than in normal cells. In particular, the final product 2c (IC_50_ = 12.19 ± 1.52 μM) showed more potent anti-tumor activity against A549 cells. It was also superior to formononetin and 5-FU. To identify the potential biological targets, the core-expressed gene *SHMT2* in lung cancer mitochondria was screened using network pharmacology technology, and molecular docking analysis confirmed the stable binding of the end products to the amino acid residues of the core genes through the formation of hydrogen bonds and via other interactions. In addition, molecular docking simulations further confirmed that the end product exhibited excellent stability when bound to *SHMT2*. These results suggest that triphenylphosphine-containing formononetin derivatives are worthy of further exploration in the search for novel drug candidates for the treatment of cancer.

## 1. Introduction

Mitochondria serve as central hubs for metabolic and signaling regulation in tumor cells, playing multifaceted roles in cancer development and progression. Metabolic reprogramming is characterized by the coordinated enhancement of both oxidative phosphorylation (OXPHOS) and glycolysis, while mutations in TCA cycle enzymes (e.g., IDH, SDH) drive the accumulation of oncogenic metabolites. Furthermore, aberrant mitochondrial dynamics (fusion/fission imbalance) and dysregulated ROS homeostasis have been shown to promote malignant phenotypes [1]. Recent studies have demonstrated that *DDX5* supports small-cell lung cancer progression by maintaining respiratory chain function, whereas Supinoxin induces metabolic collapse through the inhibition of the *DDX5*-ROS axis, suggesting mitochondrial dysfunction as a novel therapeutic target in efforts to overcome chemoresistance [2]. Targeted therapy entails the design and synthesis of drugs based on the molecular and cellular disparities between tumor and normal cells, specifically aiming at known oncogenes [3,4]. Among various delivery strategies, the mitochondrial accumulation of such agents has emerged as one of the most effective approaches to enhance anti-tumor efficacy while minimizing systemic toxicity [5]. Current targeting strategies include (1) nanoparticle-mediated mitochondrial drug delivery systems, (2) inhibitors of electron transport chain complexes (e.g., IACS-010759 targeting Complex I), (3) interventions in metabolic enzymes (e.g., the glutaminase GLS inhibitor CB-839), and (4) precision therapies utilizing mitochondrial-penetrating peptides [6]. Due to the differences in mitochondrial membrane potential between malignant and normal cells, numerous lipophilic cations preferentially accumulate within the mitochondria of tumor cells. These cations exert proapoptotic effects by inhibiting the electron transport chain, disrupting mitochondrial membrane potential, and triggering the opening of membrane permeability transition pores [7,8]. Representative lipophilic cations include triphenylphosphine (TPP) [9,10], berberine [11], rhodamine, and heptamethine cyanine dyes. Among these, TPP is noted for its superior lipophilicity and enhanced ability to traverse the mitochondrial membrane compared to other cations [12,13]. To exploit this property, numerous mitochondria-targeted anti-tumor derivatives have been synthesized by conjugating TPP to bioactive small molecules via ester bonds. Examples include derivatives glycyrrhizic acid, betulinic acid, ursolic acid, oleanolic acid, etc., all of which demonstrate improved potency and selectivity as a result of TPP conjugation. Accordingly, mitochondrial-targeting modification via TPP linkage represents a promising strategy for the structural optimization of natural-product-based therapeutics [14,15,16,17,18,19].

Formononetin, an isoflavonoid present in red plantain, astragalus, and soy, and its anti-tumor and neuroprotective activities have received increasing attention in recent years. However, due to the chemical structure of formononetin, it has poor aqueous solubility, and it is mainly absorbed in the intestinal tract after oral administration. It is quickly metabolized to form glucuronic acid or sulphuric acid conjugates. This, to some extent, counteracts the pharmacological activity of the hydroxyl groups that are supposed to be present [20]. Therefore, many researchers have made structural modifications, mainly to the 7-hydroxyl group, to enhance anti-tumor activity. Formononetin is not only a starting material used to prepare effective and biologically active compounds, but it also improves the sustainability of the overall drug design [21].

Accordingly, formononetin was structurally modified at the 7-positon by introducing triphenylphosphonium (TPP^+^) moieties through ester linkages of varying alkyl chain lengths. A total of 21 derivatives were synthesized and evaluated for their cytotoxic effects against four tumor cell lines and a normal cell line using the MTT assay. Furthermore, molecular docking and molecular dynamics simulations were performed for the selected lead compound, 2c, to investigate its binding interactions with serine hydroxymethyltransferase 2 (*SHMT2*), a mitochondrial protein differentially expressed in lung cancer.

## 2. Results

### 2.1. Synthesis

**Figure 1:**
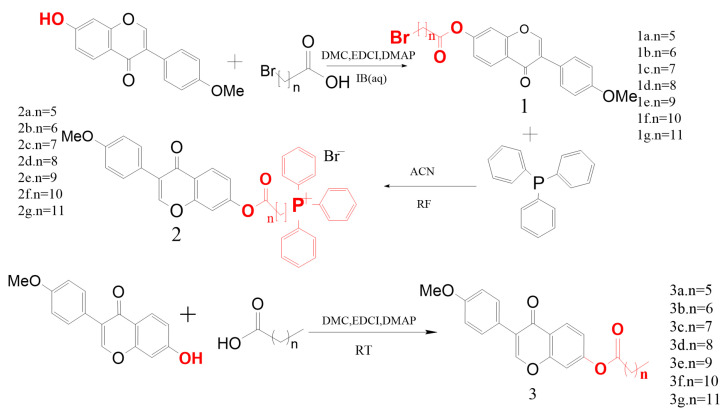
Synthesis process of target derivatives.

**Figure 2:**
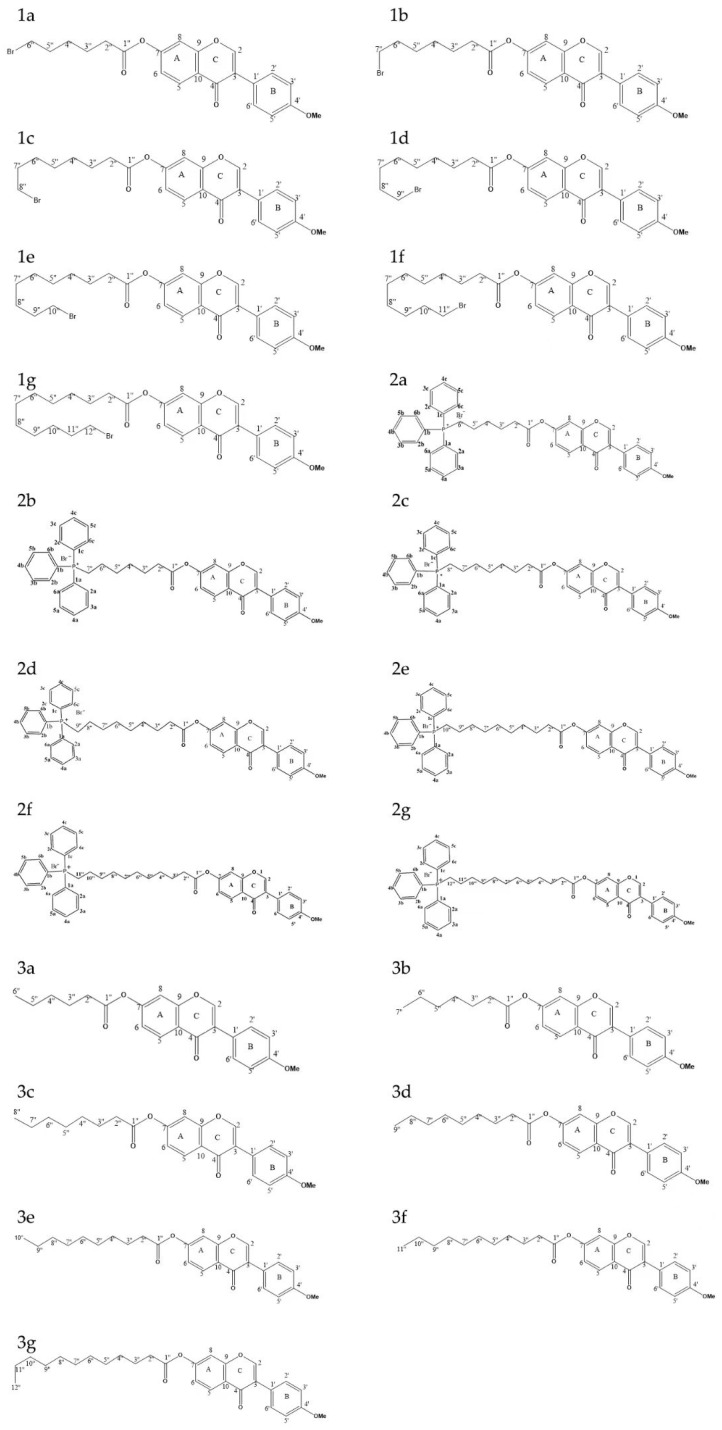
Structural diagrams of 21 derivatives.

Seven alkyl chains of varying lengths were selected as linkers for chemical synthesis (Figure 1). The synthesized compounds, 1a~1g, 2a~2g, and 3a~3g, were characterized and confirmed using ^1^H-NMR and ^13^C-NMR spectroscopy (Figure 2).

Compared with formononetin, the intermediates and fatty acids exhibited 6–12sets of carbon–hydrogen signals in the ^1^H-NMR spectrum at 3.85 ppm (-OCH_3_) and in the ^13^C-NMR spectrum at 55.44 ppm (-OCH_3_), respectively. Furthermore, the original carbon signal at 7-position (164.76 ppm) shifted downfield by an average of 10.16 ppm, indicating that the esterified alkyl chain was successfully introduced at the C-7 position of formononetin. Compared with the intermediate, the final product displayed 9 and 6 sets of additional hydrogen signals between 7.83 and 7.63 ppm, along with 15 sets of carbon signals in the ^13^C-NMR spectrum around 135.11–130.65 ppm and 119.58 ppm. Moreover, the terminal carbon signals exhibited splitting, confirming the successful substitution of triphenylphosphine in the alkyl chain.

### 2.2. MTT Results

To evaluate the tumor-inhibitory and selective cytotoxic effects of the synthesized compounds across a range of concentrations, intermediates (1a~1g), final products (2a~2g), and fatty acids esters (3a~3g) were tested against four human tumor cells lines (HGC-27, MCF-7, A549, PC-3M) as well as the normal human embryonic kidney cell line (HEK-293). Forminonetin, 5-fluorouracil (5-FU), and doxorubicin (DOX) were included as positive control groups, as shown in Table 1. Compared to formononetin (IC_50_ = 83.02 ± 6.25 μM) and 5-FU (IC_50_ = 22.92 ± 3.54 μM), the final product 2c (IC_50_ = 12.19 ± 1.52 μM) exhibited significantly enhanced anti-tumor activity against A549 cells, indicating that substitution at the 7-position in hydroxyl group of formononetin significantly influenced anti-tumor activity. Furthermore, 2c showed the most potent cytotoxicity against HGC-27 cells (IC_50_ = 18.62 ± 1.60 μM). The final derivatives (2a~2g) generally exhibited strong anti-tumor activity, while FMN and 5-FU both showed limited efficacy against MCF-7 cells, with IC_50_ values exceeding 100 μmol/L. Similarly, most of the fatty acid esters (3a-3g) and intermediates (1a~1g) displayed IC_50_ greater than 100 μmol/L in the PC-3M cell line, suggesting weak or negligible activity. The final product 2c (IC_50_ = 21.73 ± 1.25 μM) had superior anti-tumor activity compared to FMN (IC_50_ = 76.39 ± 6.47 μM) and 5-FU (IC_50_ = 48.05 ± 3.76 μM). However, some compounds showed no appreciable anti-tumor activity. Although multiple concentrations were evaluated in this study, certain derivatives failed to reach the half-maximal inhibitory concentration (IC_50_), suggesting limited inhibitory activity on cell viability within the tested concentration range. The final products (2a~2g) demonstrated variable inhibitory activity against the four tumor cell lines compared to formononetin, fatty acids (3a~3g), and intermediates (1a~1g). Notably, formononetin-containing triphenylphosphine exhibited lower cytotoxicity toward normal cells, indicating a degree of selectivity for cancer cells. Among the tested cancer cell lines, human non-small-cell lung cancer A549 showed relatively higher sensitivity to the final products. Consequently, the final product 2c was selected for further studies in A549 cells (Table 1).

### 2.3. Network Pharmacology Results

#### 2.3.1. Tumor Mitochondrial Differentially Expressed Gene Analysis Results

**Figure 3:**
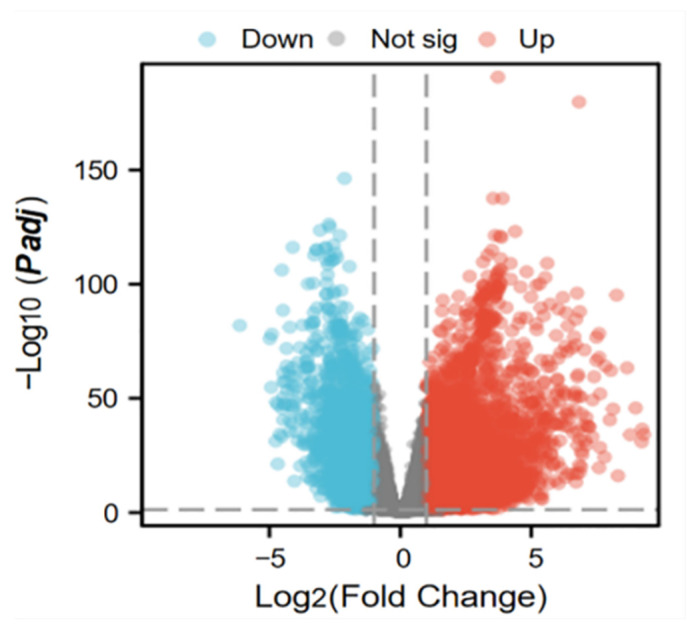
Volcano map of differentially expressed genes in tumors.

**Figure 4:**
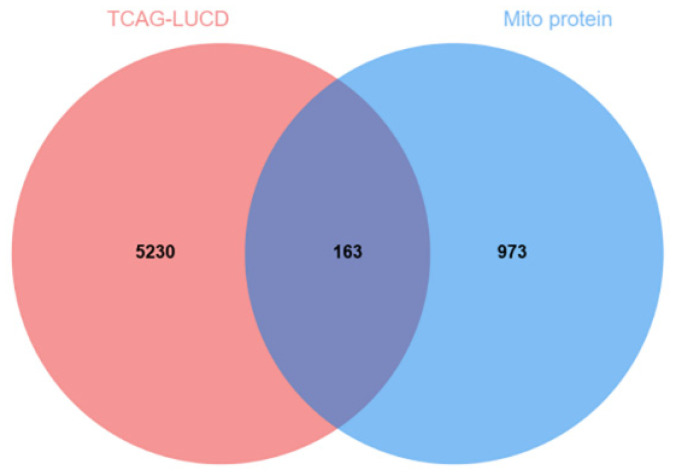
Tumor and mitochondrial intersection genes.

A total of 5395 differentially expressed genes (DEGs) between lung adenocarcinoma samples and normal controls were retrieved from the TCGA database. A volcano plot was generated using the “Ggplot2” package to visualize these DEGs (Figure 3). In the plot, each dot represents a gene: red indicates upregulation genes, blue denotes downregulation genes, and gray corresponds to genes without statistically significant changes. Notably, the number of upregulation genes substantially exceeded that of downregulation genes. Subsequently, 5393 tumor-related DEGs were intersected with a set of 1136 mitochondrial protein-coding genes using a Venn diagram analysis. This resulted in the identification of 163 differentially expressed mitochondrial genes in lung cancer (Figure 4).

#### 2.3.2. Results of GO and KEGG Pathway Enrichment Analysis of Intersected Genes

**Figure 5:**
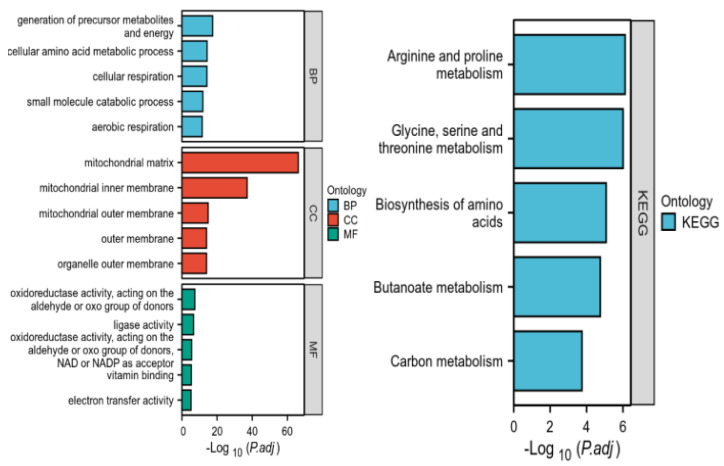
Enrichment analysis of genes intersecting tumors and mitochondria.

We performed GO enrichment analysis and KEGG pathway enrichment analysis of the 163 intersected genes using R language (Figure 5). The functional impact of differentially expressed mitochondrial genes in lung cancer was examined through biological processes (BPs), molecular functions (MFs), and cellular components (CCs) associated with this gene set. In the BP analysis, the DEGs were primarily involved in the biosynthesis and accumulation of primary metabolites, cellular amino acid metabolic processes, cellular respiration, etc. The CC analysis revealed that these DEGs were mainly associated with the mitochondrial matrix, inner membrane, and outer membrane. The molecular function (MF) analysis revealed the significant enrichment of three key biological activities among the differentially expressed genes (DEGs):(1) oxidoreductase activity, utilizing aldehyde or oxygen groups as electron donors, (2) vitamin binding with NAD/NADP as cofactor acceptors, and (3) electron transfer activity. These functional associations were identified as being statistically significant (*p* < 0.05, FDR-corrected) through comprehensive bioinformatics analysis. According to the KEGG pathway enrichment analysis, the DEGs were predominantly enriched in arginine and proline metabolism, glycine, serine and threonine metabolism, and carbon metabolism.

#### 2.3.3. Core Gene Screening and Protein–Protein Interaction (PPI) Network Construction Results

**Figure 6:**
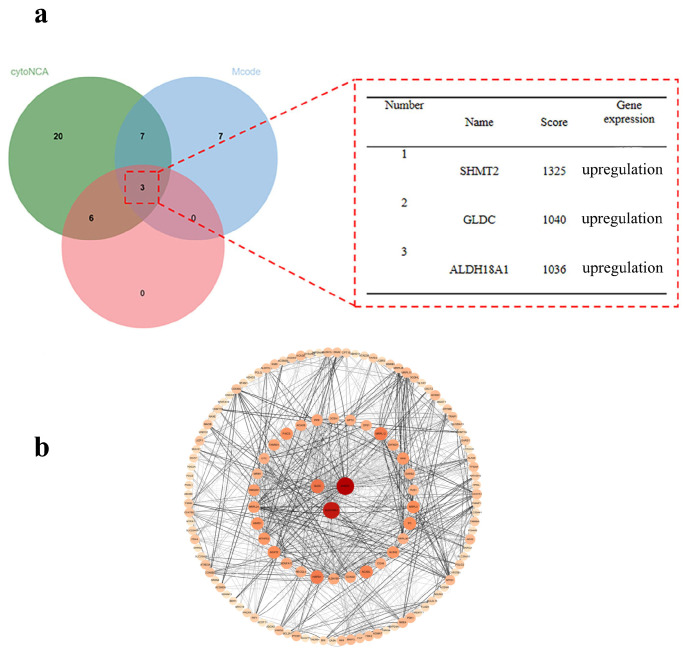
(**a**) Nuclear geneveen plot; (**b**) nuclear gene PPI plot.

The 163 overlapping DEGs were screened using three analytical methods—CytoNCA, Mcode, and Cytohubba—based on data from STRING database. As shown in Figure 6, the three methods identify the top three hub genes, *SHMT2*, *GLDC*, and *ALDH18A1*, with scores of 1325, 1040, and 1036, respectively, all of which were upregulated in that order. The PPI network illustrated the interactions between 163 genes and ranked them according to their degree values. In the figure, node size and color intensity correspond to degree values: the larger and darker the nodes, the higher the degree value. Genes with higher degree values are considered more likely to be core targets. Therefore, *SHMT2*, the top-ranked core gene, was selected for molecular docking prediction analysis.

### 2.4. Molecular Docking Results

**Figure 7:**
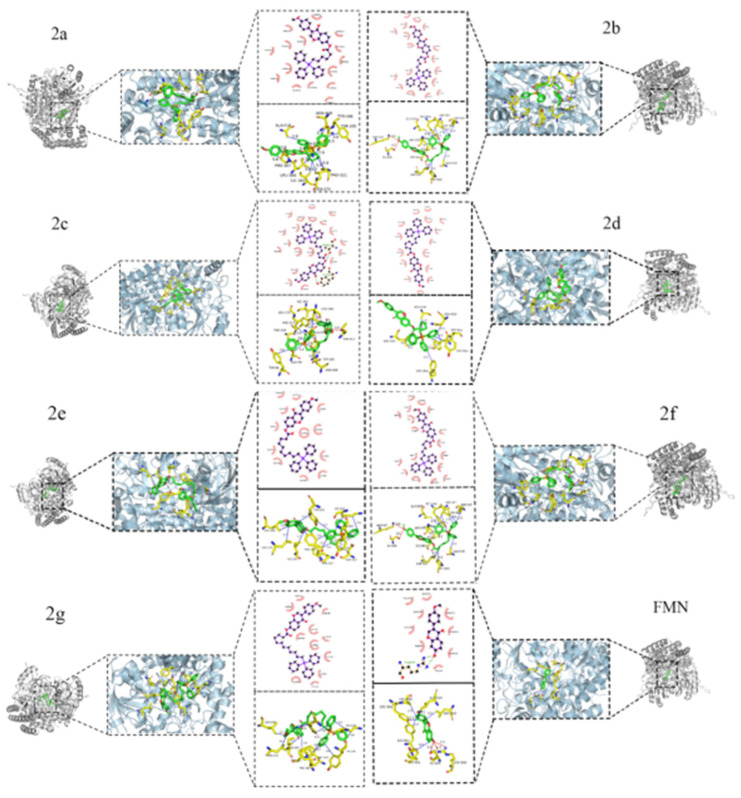
Two-dimensional and three-dimensional interactions of end products 2a~2g and formononetin.

Molecular docking analysis was performed to confirm the interaction between the final compounds and the *SHMT2* protein (PDB ID:8GKT), as shown in Table 2 and Figure 7. The results demonstrated that the binding energies of the derivatives were all lower than that of the parent compound, formononetin, suggesting that the triphenylphosphine-based small molecule derivatives exhibit potential biological activity. The final compounds exhibited variations in the types and numbers of amino acid residues interacting with 8GKT. Notably, TYR (-C_6_H_4_OH, a side-chain benzene ring with a hydroxyl group) was involved in phosphorylation reactions, LEU (-CH_2_-CH(CH_3_)_2_, a side-chain isobutyl group) formed part of the hydrophobic core of the protein, and ILE (-CH(CH_3_)-CH_2_-CH_3_, a side-chain isobutyl group) participated in protein structural formation. These three residues appeared across multiple derivatives and docking complexes, suggesting a dominant role in ligand–protein binding. In addition, GLU (-CH_2_-CH_2_-COOH, a side-chain carboxyl group), TYR (-C_6_H_4_OH, a side-chain benzene ring with one hydroxyl group), and THR (-CH(OH)-CH_3_ a side-chain hydroxyethyl group) were uniquely involved in binding with the final compound 2c. Among them, THR-411, due to its polarity, was particularly important as it contributed to hydrogen bond formation, which is essential for the specific binding of the final compound 2c to *SHMT2*. This interaction is critical for the activity and potential catalytic role of the *SHMT2* protein.

### 2.5. Molecular Dynamics Simulation Results

**Figure 8:**
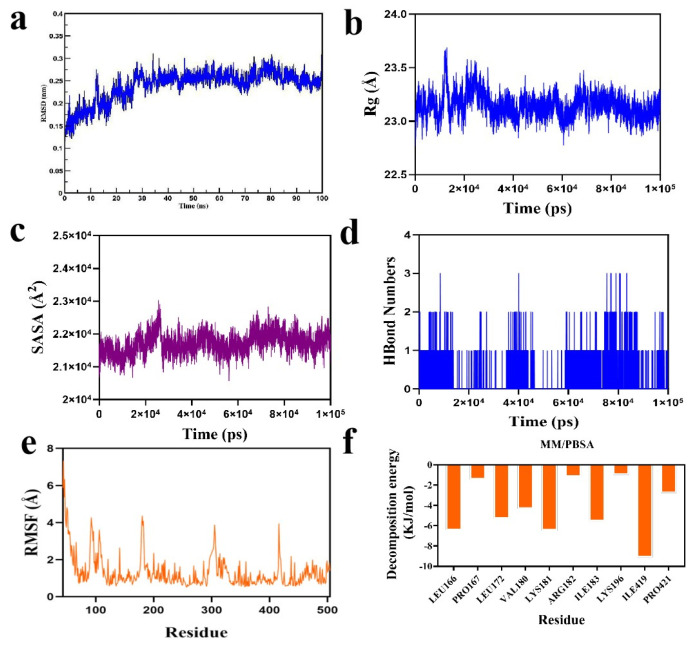
Molecular dynamics simulation analysis of final product 2c in complex SHMT2 protein: (**a**) RMSD of protein and ligand; (**b**) Rg of protein and ligand; (**c**) SASA of protein and ligand; (**d**) number of hydrogen bonds between protein and ligand; (**e**) RMSF of protein and ligand; (**f**) MM-PBSA of protein and ligand.

As shown in Figure 8a, the complex system reached equilibrium after 20 ns and subsequently fluctuated around 2.6 Å, indicating that the small molecule exhibits high stability upon binding to the target protein. Further analysis revealed that the radius of gyration (Rg) values of the complex systems in Figure 8b,c showed only slight fluctuations, along with the solvent-accessible surface area (SASA) during the simulation. This indicates that the protein-small molecule complexes underwent minor conformational changes over time. The number of hydrogen bonds formed between the small molecules and the target proteins during the simulation is shown in Figure 8d; typically, the complexes have two hydrogen bonds, with the overall range varying from 0 to 3, indicating that the protein–ligand complexes exhibited favorable hydrogen bonding interactions. As shown in Figure 8e, the root mean square fluctuation (RMSF) values of the complex system components were relatively low (mostly below 4 Å), suggesting limited flexibility and enhanced structural stability.

Subsequently, based on the binding conformation of the compound, the binding free energy between the small molecule and the target protein was calculated using the MM/PBSA method. The binding free energy of the compound system was determined to be −93.863 kJ/mol. The negative value indicates that the molecule exhibits binding affinity toward the target protein, with lower values corresponding to stronger binding. Therefore, the compound system demonstrates a relatively high binding affinity. Further analysis was conducted to identify the amino acid residues that significantly contributed to the binding of the small molecules within the compound. The results revealed that residues ILE-419, ILE-183, LYS-181, LEU-166, LEU-172, and VAL-180 contributed notably to the binding (Figure 8f). Among these, the ILE and LEU residues not only showed the largest contribution values but also played critical roles in the molecular docking process. This suggests that these amino acid residues may be essential in the catalytic mechanism.

In summary, the small molecule binds stably to the target protein, exhibits low binding free energy, and forms favorable hydrogen bonding interactions within the compound. Therefore, it is likely that the small molecule will exert its biological effects by inhibiting the target protein.

## 3. Discussion and Future Perspectives

The behavior of cancer cells stems from the uncontrolled and continuous proliferation of normal cells, which contributes to the high morbidity and mortality associated with tumors [22]. The growth, metastasis, and invasion of cancer depend on mitochondrial respiration [23]. Although most cancer cells rely on glycolytic metabolism, mitochondria play a functional and important role in cell survival and proliferation in many types of cancer [24,25,26,27]. At the same time, the limited specificity of current pharmacological inhibitors in cancer therapy often leads to severe adverse effects. As a result, enhancing drug bioavailability and optimizing mitochondrial-targeting strategies, particularly through the development of innovative drug delivery systems, has emerged as a major focus in recent oncological research [28].

The mitochondrial membrane exhibits an inherent negative surface charge, while triphenylphosphine-conjugated drug derivatives possess a delocalized positive charge. Owing to the membrane potential between triphenylphosphine and the inner electrochemical potential gradient, these positively charged derivatives preferentially accumulate on the mitochondria surface, where the negative potential of the matrix is substantially greater than that of the cytosolic side. The substantial membrane potential difference (Δψm) across the mitochondrial inner membrane consequently establishes a robust electrochemical gradient, driving the selective accumulation of triphenylphosphonium (TPP)-conjugated drug derivatives at concentrations up to 10-fold higher than cytosolic levels. The resulting eletrostatic force facilitates the transmembrane transport of the derivatives, leading to their accumulation within the mitochondria and enabling a mitochondria-targeting effect [29,30,31]. Such derivatives not only inhibit mitochondrial respiration but also reduce cellular ATP, preventing the efflux of compounds through resistance proteins and thereby enhancing anti-tumor activity [32]. This targeted delivery strategy represents a promising approach for the development of more effective cancer therapeutics.

Formononetin is a naturally occurring isoflavonoid that has demonstrated notable inhibitory activity on cancer cell proliferation [33]. Due to its low solubility and high permeability, formononetin is classified as a poorly soluble, highly permeable drug, which significantly limits its absorption and is one of the main factors contributing to its low bioavailability. To overcome this limitation and fully exploit the anti-tumor potential of formononetin, more and more research has focused on structural modification strategies aimed at enhancing its bioactivity and pharmacokinetic impact in terms of anti-tumor activity [34].

For instance, Hong-Yan Lin et al. [35] synthesized a series of formononetin derivatives by utilizing the 7-position of the molecule as the modification site. These derivatives demonstrated potent inhibitory activity against breast cancer cell lines. In the present study, a similar approach was adopted by introducing a pharmacophore group at the 7-position of formononetin to assess whether such modifications could significantly enhance its biological activity and improve mitochondrial-targeting capability.

Fangfei Liu et al. conjugated triphenylphosphine to burdock sapogenins using bromo-acid with varying alkyl chain lengths and observed that the length of the alkyl chains significantly influenced the tumor-inhibitory activity across different tumor cell lines [35]. Based on these findings, we investigated the structure–activity relationship (SAR) of the target derivatives by systematically modifying the alkyl chain length at the 7-position of formononetin. Previous studies have reported that the esterification yield of formononetin remains below 50% when anhydrous tetrahydrofuran or pyridine is employed as the reaction solvent, regardless of whether the reaction is performed under ice-bath or heating conditions [36,37,38,39]. To improve yield, dichloromethane was selected as the reaction solvent in our study, and a substantially higher conversion rate was achieved when the reaction was conducted under ice-bath conditions for 7–10 h.

To minimize the formation of by-products during the synthesis of intermediates, a molar ratio of formononetin to bromo-acid of 1:2.5 was employed. In the subsequent step, it was noted that the use of excessive triphenylphosphine complicated the purification process due to its incomplete removal. Furthermore, prolonged refluxing led to excessive water vapor in the reaction vessel, which could hydrolyze the ester bond, resulting in a markedly reduced yield of the final compound.

Therefore, using procedures reported in the previous studies [40,41], we employed a molar ratio of 1:3 between the intermediate and triphenylphosphine. The reaction solvent was changed from acetone to anhydrous acetonitrile, and the reaction was performed under reflux conditions for 40–48 h, resulting in a notably higher product yield. For the synthesis of fatty acid esters, dichloromethane was employed as the reaction solvent, and a 1.5-fold molar excess of the corresponding acid was added. The reaction was conducted over a 1–4 h period, which enabled the complete conversion of formononetin and maximum yield. Although the equilibrium solubility and oil–water partition coefficients of the synthesized formononetin derivatives were not measured in this study, it was observed during the experimental process that formononetin exhibited almost no solubility at room temperature in a range of solvents, including water, methanol, ethanol, acetone, tetrahydrofuran, ethyl acetate, dichloromethane, and chloroform. In contrast, the modified formononetin derivatives (2a~2g) demonstrated improved solubility in solvents such as acetate, tetrahydrofuran, ethyl acetate, dichloromethane, and chloroform. The introduction of triphenylphosphine in the solubility of formononetin to a certain extent.

The newly synthesized derivatives were characterized by NMR spectroscopy and subsequently evaluated for cytotoxic activity using the MTT assay. Formononetin, 5-FU, and DOX were employed as positive controls. Overall, the final products (2a~2g) exhibited superior or comparable growth-inhibitory activity relative to formononetin across the tested cancer cell lines. Among them, derivatives 2a, 2c, 2f, and 2g demonstrated notably enhanced cytostatic activity. In particular, 2c exhibited more potent growth-inhibitory activity than formononetin and 5-FU against all three cancer cell lines tested.

Specifically, in the case of HGC-27, the IC_50_ values of derivatives 2b, 2d, 2e, 2f, and 2g were 32.12 μM, 29.14 μM, 39.16 μM, 30.43 μM, and 29.36 μM, respectively. These values were comparable to those of formononetin (IC_50_ = 29.55 μM) and 5-FU (IC_50_ = 36.24 μM), indicating similar or slightly improved inhibitory activity. Notably, derivatives 2a and 2c exhibited growth-inhibitory activities against HGC-27 cells that were more than twofold greater than those of formononetin and 5-FU. For MCF-7 cells, derivatives 2a and 2g exhibited better cytotoxic effects than those of formononetin and 5-FU, with derivative 2c demonstrating the most pronounced growth inhibition. Overall, the IC_50_ values of this series of derivatives were lower than those of both formononetin and 5-FU, suggesting improved anti-proliferative potency.

For A549 cells, the IC_50_ values of derivatives 2a~2g were 28.79 μM, 42.22 μM, 12.19 μM, 31.21 μM, 38.96 μM, 20.61 μM, and 23.64 μM, respectively. All values were notably lower than those of formononetin (IC_50_ = 83.02 μM), but not as low as those of 5-FU (IC_50_ = 22.92 μM). Among them, derivatives 2c, 2f, and 2g demonstrated particularly potent activity, with IC_50_ values not only significantly lower than that of formononetin, representing approximately a 7-fold increase in cytostatic effect, but also exceeding the inhibitory activity of 5-FU by more than 1.5-fold.

For PC-3M cell lines, derivatives 2c, 2f, and 2g exhibited IC_50_ values of 21.73 μM, 27.86 μM, and 41.16 μM, respectively, indicating greater cytotoxicity compared to formononetin (IC_50_ = 76.39 μM) and 5-FU (IC_50_ = 48.05 μM). In contrast, the remaining derivatives showed IC_50_ values exceeding that of 5-FU against PC-3M cells, indicating that although they exhibited some degree of growth-inhibitory activity against PC-3M cells, their efficacy was inferior to that of 5-FU.

For HEK-293 cell lines, derivatives 2a, 2c, 2f, and 2g, which exhibited potent inhibitory activity on cancer cell lines, also demonstrated some degree of cytostatic activity. Among these, derivative 2c showed the weakest growth-inhibitory activity on HEK-293 cells, indicating relatively lower toxicity toward normal cells compared to formononetin.

In contrast, the corresponding intermediate (1a~1g) and fatty acid ester controls (3a~3g), which share the same alkyl chain length as derivatives 2a-2g, exhibited reduced or negligible growth-inhibitory against tumor cells compared to formononetin, and did not display notable tumor selectivity. In summary, the influence of C-7 alkyl chain substituents on formononetin derivatives was systematically evaluated with respect to cytotoxicity. The biological activity of these derivatives was found to be significantly affected by carbon chain length. When the alkyl chain was short, the spatial conformation of formononetin was perturbed by the lipophilic cation 2a, potentially hindering the accessibility of its effector groups. Conversely, derivatives with longer carbon chains exhibited reduced structural stability, which may have indirectly compromised the functionality of formononetin’s effector groups due to excessive chain extension, ultimately leading to diminished activity. The presence of the lipophilic cation triphenylphosphine improves the solubility of formononetin compared to fatty acids and intermediates, and the positively charged nature of the cation leads to greater aggregation in mitochondria. Therefore, it was concluded that triphenylphosphine derivatives with a carbon chain length of 7–8 carbon atoms could further enhance the bioactivity of formononetin. Among all synthesized compounds, derivative 2c exhibited the most potent anti-tumor activity against A549 cells and was consequently selected for further investigation.

The mitochondrial core gene *SHMT2* was identified as being differentially expressed in lung cancer through network pharmacology analysis. Mitochondrial serine hydroxymethyltransferase (*SHMT2*) is primarily localized in the mitochondrial matrix and plays a critical role in maintaining standard methylation patterns, genomic stability, and genetic variation. *SHMT2* is a key enzyme involved in serine metabolism, catalyzing the conversion of serine into glycine and a one-carbon unit (CH_2_-THF) [42,43,44]. The glycine produced through this reaction can trigger apoptosis by altering mitochondrial membrane permeability and increasing reactive oxygen species (ROS) production [45]. *SHMT2* has been shown to play a pivotal role in various cancers. Its overexpression promotes cell proliferation, invasion, and tumorigenesis, whereas *SHMT2* knockdown has been reported to inhibit tumor progression [46]. For instance, the silencing of *SHMT2* suppresses the expression of HIF1α, thereby reducing the proliferative capacity of malignant tumors [47]. Moreover, the inhibition of *SHMT2* activity through AKT pathway suppression has been associated with reduced metastatic potential in thyroid-like carcinoma [48]. Thus, *SHMT2* represents a promising therapeutic target and a valuable tumor prognostic biomarker in oncology.

In vivo experiments cannot be conducted at this stage. Future efforts will focus on the following aspects: 1. the optimization of in vitro mechanisms—additional in vitro mechanistic studies will be conducted to further enhance the content of this research; 2. the establishment of animal models—either A549 xenograft nude mouse models or tumor-transplanted zebrafish models will be developed to evaluate the drug’s tumor inhibition rate, toxicity profile, and metabolic characteristics in vivo.

## 4. Materials and Methods

### 4.1. Chemical Reagents

The formononetin dioxide (purity ≥ 98%) used in this study was provided by Chengdu Sodium Columbium Biotechnology Co., Ltd. (Chengdu, China). All chemical reagents required for the chemical synthesis experiments were provided by Xi Long Science Co., Ltd. (Guangzhou, China), and the chemical synthesis drugs were provided by McLean Biochemical Technology Co., Ltd. (Shanghai, China). Cell culture-related reagents, including RPMI-1640 medium, phosphate-buffered saline (PBS), trypsin, fetal bovine serum (FBS), and a penicillin/streptomycin (SP) mixture, were provided by Gibco (Carlsbad, CA, USA). 5-Fluorouracil (5-FU) and dimethyl sulfoxide (DMSO) were provided by Shanghai Aladdin Biochemical Science and Technology Co., Ltd. (Shanghai, China). The MTT thiazolyl blue reagent was provided by Beijing Solepol Biotechnology Co., Ltd. (Beijing, China).

### 4.2. Design and Synthesis of the Derivatives

In the present study, 21 derivatives were chemically synthesized as follows:

First, 40 mg of formononetin was placed in a reaction vial containing 15 mL of dichloromethane. The corresponding bromo-acid (6-bromohexanoic~12-bromododecanoic acid), EDCI, and DMAP were added in a molar ratio (formononetin: bromo-acid: EDCI: DMAP = 1:2.5:5:2) and allowed to dissolve completely in dichloromethane. The reaction was kept at 0 °C for 7–10 h. TLC confirmed that the reaction was complete. The solvent was recovered under reduced pressure, and the clarified solution became white crystals. Purification was performed by silica gel column chromatography (wet column loading, dry sampling). Separation conditions were as follows: petroleum ether (60–90 °C): acetone = 10:1–14:1. The collected eluate was detected by TLC, and then the target solvent was recovered under reduced pressure to obtain intermediates of different chain lengths (1a~1g). The intermediates (1a~1g) were separately placed in a reaction vial containing 15 mL of acetonitrile to add triphenylphosphine according to the molar ratio (intermediate: triphenylphosphine = 1:3). They underwent reflow in acetonitrile for 40–48 h. The reaction was confirmed to be complete by TLC, after which the clarified solution was obtained. The solvent was recovered under reduced pressure, and the clarified solution was crystallized. Separation and purification were performed via silica gel chromatography (column wet loading, sample wet loading). Separation conditions were as follows: trichloromethane: methanol = 10:1. Then, we performed TLC detection of the collected eluent and the substitution of triphenylphosphine for bromine on the alkyl chain to obtain the final product (2a~2g).

7-O-(6-bromohexanoyl)-formononetin (1a) was synthesized from formononetin and 6-bromohexanoic acid with a yield of 85.99%.

^1^H-NMR (300.00 MHz, CDCl_3_) *δ*_ppm_:8.33 (d, J = 8.7 Hz, 1H, H-5), 7.98 (s, 1H, H-2), 7.52–7.48 (m, 2H, H-2′, 6′), 7.30 (d, J = 2.1 Hz, 1H, H-8), 7.16 (dd, J = 8.7, 2.1 Hz, 1H, H-6), 7.00–6.95 (m, 2H, H-3′, 5′), 3.85 (s, 3H, 4′-OMe), 3.45 (t, J = 6.6 Hz, 2H, H-6″), 2.65 (t, J = 7.5 Hz, 2H, H-2″), 1.99–1.90 (m, 2H, H-5″), 1.87–1.77 (m, 2H, H-3″), 1.65–1.60 (m, 2H, H-4″). ^13^C-NMR (75.00 MHz, CDCl_3_) *δ*_ppm_:175.87 (C-4), 171.19 (C-1″), 159.84 (C-4′), 156.77 (C-9), 154.54 (C-7), 152.73 (C-2), 130.22 (C-2′, 6′), 127.96 (C-5), 125.28 (C-1′), 123.92 (C-3), 122.40 (C-6), 119.53 (C-10), 114.15 (C-3′, 5′), 110.96 (C-8), 55.44 (-OCH_3_), 34.24 (C-2″), 33.49 (C-6″), 32.40 (C-5″), 27.66 (C-4″), 24.02 (C-3″).

7-O-(7-bromoheptanoyl)-formononetin (1b) was synthesized from formononetin and 7-bromoheptanoic acid with a yield of 94.32%.

^1^H-NMR (300.00 MHz, CDCl_3_) *δ*_ppm_:8.32 (d, J = 8.7 Hz, 1H, H-5), 7.98 (s, 1H, H-2), 7.52–7.47 (m, 2H, H-2′, 6′), 7.29 (d, J = 2.1 Hz, 1H, H-8), 7.15 (dd, J = 8.7, 2.1 Hz, 1H, H-6), 7.00–6.96 (m, 2H, H-3′, 5′), 3.84 (s, 3H, 4′-OMe), 3.43 (t, J = 6.6 Hz, 2H, H-7″), 2.63 (t, J = 7.2 Hz, 2H, H-2″), 1.95–1.86 (m, 2H, H-6″), 1.85–1.75 (m, 2H, H-3″), 1.59–1.50 (m, 2H, H-5″), 1.49–1.41 (m, 2H, H-4″). ^13^C-NMR (75.00 MHz, CDCl_3_) *δ*_ppm_:175.89 (C-4), 171.36 (C-1″), 159.83 (C-4′), 156.77 (C-9), 154.58 (C-7), 152.74 (C-2), 130.23 (C-2′, 6′), 127.95 (C-5), 125.27 (C-1′), 123.91 (C-3), 122.37 (C-6), 119.55 (C-10), 114.14 (C-3′, 5′), 110.99 (C-8), 55.48 (-OCH_3_), 34.31 (C-2″), 33.82 (C-7″), 32.57 (C-6″), 28.27 (C-4″), 27.86 (C-5″), 24.65 (C-3″).

7-O-(8-bromooctanoyl)-formononetin (1c) was synthesized from formononetin and 8-bromooctanoic acid with a yield of 96.34%.

^1^H-NMR (300.00 MHz, CDCl_3_) *δ*_ppm_:8.32 (d, J = 8.7 Hz, 1H, H-5), 7.98 (s, 1H, H-2), 7.52–7.47 (m, 2H, H-2′, 6′), 7.29 (d, J = 1.8 Hz, 1H, H-8), 7.15 (dd, J = 8.7, 2.1 Hz, 1H, H-6), 7.00–6.95 (m, 2H, H-3′, 5′), 3.84 (s, 3H, 4′-OMe), 3.42 (t, J = 6.9 Hz, 2H, H-8″), 2.62 (t, J = 7.5 Hz, 2H, H-2″), 1.93–1.86 (m, 2H, H-7″), 1.83–1.74 (m, 2H, H-3″), 1.48–1.42 (m, 4H, H-4″, 5″), 1.39–1.33 (m, 2H, H-6″). ^13^C-NMR (75.00 MHz, CDCl_3_) *δ*_ppm_:175.92 (C-4), 171.45 (C-1″), 159.83 (C-4′), 156.78 (C-9), 154.62 (C-7), 152.71 (C-2), 130.23 (C-2′, 6′), 127.95 (C-5), 125.28 (C-1′), 123.92 (C-3), 122.35 (C-6), 119.57 (C-10), 114.15 (C-3′, 5′), 110.00 (C-8), 55.44 (-OCH_3_), 34. (C-2″), 33.96 (C-8″), 32.78 (C-7″), 28.97 (C-4″), 28.50 (C-5″), 28.06 (C-6″), 24.75 (C-3″).

7-O-(9-bromononanoi)-formononetin (1d) was synthesized from formononetin and 9-bromononanoic acid with a yield of 80.50%.

^1^H-NMR (300.00 MHz, CDCl_3_) *δ*_ppm_:8.32 (d, J = 8.7 Hz, 1H, H-5), 7.98 (s, 1H, H-2), 7.53–7.48 (m, 2H, H-2′, 6′), 7.29 (d, J = 2.1 Hz, 1H, H-8), 7.16 (dd, J = 8.7, 2.1 Hz, 1H, H-6), 7.00–6.95 (m, 2H, H-3′, 5′), 3.84 (s, 3H, 4′-OMe), 3.42 (t, J = 6.6 Hz, 2H, H-9″), 2.62 (t, J = 7.5 Hz, 2H, H-2″), 1.92–1.82 (m, 2H, H-8″), 1.80–1.73 (m, 2H, H-3″), 1.48–1.33 (m, 8H, H-4″, 5″, 6″, 7″). ^13^C-NMR (75.00 MHz, CDCl_3_) *δ*_ppm_:175.91 (C-4), 171.50 (C-1″), 159.84 (C-4′), 156.79 (C-9), 154.65 (C-7), 152.75 (C-2), 130.21 (C-2′, 6′), 127.91 (C-5), 125.28 (C-1′), 123.93 (C-3), 122.35 (C-6), 119.58 (C-10), 114.15 (C-3′, 5′), 110.95 (C-8), 55.43 (-OCH_3_), 34.46 (C-2″), 34.05 (C-9″), 32.85 (C-8″), 29.15 (C-6″), 29.05 (C-5″), 28.66 (C-4″), 28.18 (C-7″), 24.83 (C-3″).

7-O-(10-bromodecanoyl)-formononetin (1e) was synthesized from formononetin and 10-bromodecanoic acid with a yield of 76.14%.

^1^H-NMR (300.00 MHz, CDCl_3_) *δ*_ppm_:8.33 (d, J = 9.3 Hz, 1H, H-5), 7.98 (s, 1H, H-2), 7.53–7.48 (m, 2H, H-2′, 6′), 7.29 (d, J = 1.5 Hz, 1H, H-8), 7.16 (dd, J = 8.7, 2.4 Hz, 1H, H-6), 7.01–6.96 (m, 2H, H-3′, 5′), 3.85 (s, 3H, 4′-OMe), 3.42 (t, J = 6.9 Hz, 2H, H-10″), 2.61 (t, J = 7.8 Hz, 2H, H-2″), 1.91–1.83 (m, 2H, H-9″), 1.82–1.73 (m, 2H, H-3″), 1.43–1.34 (m, 10H, H-4″, 5″, 6″, 7″, 8″). ^13^C-NMR (75.00 MHz, CDCl_3_) *δ*_ppm_:175.91 (C-4), 171.53 (C-1″), 159.85 (C-4′), 156.79 (C-9), 154.66 (C-7), 152.74 (C-2), 130.23 (C-2′, 6′), 127.94 (C-5), 125.28 (C-1′), 123.94 (C-3), 122.35 (C-6), 119.59 (C-10), 114.15 (C-3′, 5′), 110.00 (C-8), 55.44 (-OCH_3_), 34.48 (C-2″), 34.10 (C-10″), 32.90 (C-9″), 29.33 (C-5″), 29.23 (C-7″), 29.12 (C-6″), 28.80 (C-4″), 28.23 (C-8″), 24.87 (C-3″).

7-O-(11-bromoundecanoyl)-formononetin (1f) was synthesized from formononetin and 11-bromoundecanoyl acid with a yield of 86.91%.

^1^H-NMR (300.00 MHz, CDCl_3_) *δ*_ppm_:8.32 (d, J = 9.3 Hz, 1H, H-5), 7.98 (s, 1H, H-2), 7.52–7.48 (m, 2H, H-2′, 6′), 7.29 (d, J = 1.5 Hz, 1H, H-8), 7.16 (dd, J = 8.7, 2.1 Hz, 1H, H-6), 7.00–6.95 (m, 2H, H-3′, 5′), 3.85 (s, 3H, 4′-OMe), 3.41 (t, J = 6.9 Hz, 2H, H-11″), 2.61 (t, J = 7.8 Hz, 2H, H-2″), 1.90–1.81 (m, 2H, H-10″), 1.80–1.73 (m, 2H, H-3″), 1.42–1.32 (m, 12H, H-4″, 5″, 6″, 7″, 8″, 9″). ^13^C-NMR (75.00 MHz, CDCl_3_) *δ*_ppm_:175.91 (C-4), 171.56 (C-1″), 159.85 (C-4′), 156.80 (C-9), 154.68 (C-7), 152.69 (C-2), 130.24 (C-2′, 6′), 127.95 (C-5), 125.30 (C-1′), 123.95 (C-3), 122.36 (C-6), 119.59 (C-10), 114.16 (C-3′, 5′), 110.96 (C-8), 55.44 (-OCH_3_), 34.51 (C-2″), 34.13 (C-11″), 32.93 (C-10″), 29.47 (C-5″), 29.42 (C-7″), 29.30 (C-8″), 29.16 (C-4″), 28.85 (C-6″), 28.27 (C-9″), 24.89 (C-3″).

7-O-(12-bromododecanoyl)-formononetin (1g) was synthesized from formononetin and 12-bromododecanoyl acid with a yield of 85.37%.

^1^H-NMR (300.00 MHz, CDCl_3_) *δ*_ppm_:8.33 (d, J = 8.7 Hz, 1H, H-5), 7.98 (s, 1H, H-2), 7.52–7.48 (m, 2H, H-2′, 6′), 7.29 (d, J = 2.1 Hz, 1H, H-8), 7.15 (dd, J = 8.7, 2.1 Hz, 1H, H-6), 7.00–6.95 (m, 2H, H-3′, 5’), 3.85 (s, 3H, 4’-OMe), 3.41 (t, J = 6.6 Hz, 2H, H-12″), 2.61 (t, J = 7.8 Hz, 2H, H-2″), 1.90–1.81 (m, 2H, H-11″), 1.80–1.73 (m, 2H, H-3″), 1.43–1.30 (m, 14H, H-4″, 5″, 6″, 7″, 8″, 9″, 10″). ^13^C-NMR (75.00 MHz, CDCl_3_) *δ*_ppm_:176.32 (C-4), 171.98 (C-1″), 160.27 (C-4′), 157.21 (C-9), 155.10 (C-7), 153.15 (C-2), 130.66 (C-2′, 6′), 128.36 (C-5), 125.70 (C-1′), 124.37 (C-3), 122.78 (C-6), 120.01 (C-10), 114.58 (C-3′, 5′), 111.43 (C-8), 55.91 (-OCH_3_), 34.93 (C-2″), 34.57 (C-12″), 33.36 (C-11″), 29.98 (C-6″), 29.94 (C-5″/8″), 29.75 (C-9″), 29.59 (C-4″), 29.29 (C-7″), 28.70 (C-10″), 25.32 (C-3″).

7-O-(6-bromohexanoyl)-formononetin-triphenylphosphine coupling (2a) was synthesized via 1a and triphenylphosphine with a yield of 32.16%.

^1^H-NMR (300.00 MHz, CDCl_3_) *δ*_ppm_:8.27 (d, J = 8.7 Hz, 1H, H-5), 7.96 (s, 1H, H-2), 7.87–7.76 (m, 9H, H-3a, 4a, 5a, 3b, 4b, 5b, 3c, 4c, 5c), 7.72–7.66 (m, 6H, H-2a, 6a, 2b, 6b, 2c, 6c), 7.50–7.47 (m, 2H, H-2′, 6′), 7.26 (d, J = 2.1 Hz, 1H, H-8), 7.11 (dd, J = 8.7, 2.1 Hz, 1H, H-6), 6.98–6.95 (m, 2H, H-3′, 5′), 3.85 (s, 3H, 4′-OMe), 3.81 (t, J = 2.4 Hz, 2H, 6″-H-6″), 2.62 (t, J = 6.3 Hz, 2H, H-2″), 1.82–1.67 (m, 6H, 3″, H-3″, 4″, 5″). ^13^C-NMR (75.00 MHz, CDCl_3_) *δ*_ppm_:175.85 (C-4), 171.34 (C-1″), 159.75 (C-4′), 156.69 (C-9), 154.52 (C-7), 152.72 (C-2), 135.16 (4a, 4b, 4c), 133.63 (2a, 6a, 2b, 6b, 2c, 6c), 130.68 (3a, 5a, 3b, 5b, 3c, 5c), 130.17 (C-2′, 6′), 127.68 (C-5), 125.11 (C-1′), 123.90 (C-3), 122.23 (C-6), 119.60 (C-10), 118.88, 117.74 (1a, 1b, 1c), 114.08 (C-3′, 5′), 111.06 (C-8), 55.39 (-OCH_3_), 33.88 (C-2″), 29.82 (C-4″), 24.16 (C-3″), 22.91 (C-6″), 22.52 (C-5″).

7-O-(7-bromoheptanoyl)-formononetin-triphenylphosphine coupling (2b) was synthesized using 1b and triphenylphosphine with a yield of 28.01%.

^1^H-NMR (300.00 MHz, CDCl_3_) *δ*_ppm_:8.28 (d, J = 8.7 Hz, 1H, H-5), 7.97 (s, 1H, H-2), 7.88–7.79 (m, 9H, H-3a, 4a, 5a, 3b, 4b, 5b, 3c, 4c, 5c), 7.74–7.65 (m, 6H, H-2a, 6a, 2b, 6b, 2c, 6c), 7.50–7.47 (m, 2H, H-2′, 6′), 7.27 (d, J = 1.8 Hz, 1H, H-8), 7.14 (dd, J = 9.0, 2.4 Hz, 1H, H-6), 6.98–6.95 (m, 2H, H-3′, 5′), 3.85 (s, 3H, 4′-OMe), 3.81 (t, J = 5.4 Hz, 2H, H-7″), 2.60 (t, J = 7.8 Hz, 2H, H-2″), 1.76–1.66 (m, 6H, H-3″, 5″, 6″), 1.51–1.43 (m, 2H, H-4″). ^13^C-NMR (75.00 MHz, CDCl_3_) *δ*_ppm_:175.87 (C-4), 171.53 (C-1″), 159.78 (C-4′), 156.72 (C-9), 154.60 (C-7), 152.76 (C-2), 135.13 (4a, 4b, 4c), 133.80 (2a, 6a, 2b, 6b, 2c, 6c), 130.67 (3a, 5a, 3b, 5b, 3c, 5c), 130.18 (C-2′, 6′), 127.73 (C-5), 125.15 (C-1′), 123.91 (C-3), 122.24 (C-6), 119.62 (C-10), 118.98, 117.84 (1a, 1b, 1c), 114.10 (C-3′, 5′), 111.04 (C-8), 55.46 (-OCH_3_), 34.12 (C-2″), 30.06 (C-6″), 28.42 (C-4″), 24.28 (C-5″), 22.53 (C-3″), 22.99 (C-7″).

7-O-(8-bromooctanoyl)-formononetin-triphenylphosphine coupling (2c) was synthesized using 1c and triphenylphosphine with a yield of 31.6%.

^1^H-NMR (300.00 MHz, CDCl_3_) *δ*_ppm_:8.23 (d, J = 8.7 Hz, 1H, H-5), 7.96 (s, 1H, H-2), 7.83–7.73 (m, 9H, H-3a, 4a, 5a, 3b, 4b, 5b, 3c, 4c, 5c), 7.69–7.63 (m, 6H, H-2a, 6a, 2b, 6b, 2c, 6c), 7.47–7.44 (m, 2H, H-2′, 6′), 7.24 (d, J = 2.1 Hz, 1H, H-8), 7.10 (dd, J = 8.7, 2.1 Hz, 1H, H-6), 6.95–6.92 (m, 2H, H-3′, 5′), 3.80 (s, 3H, 4′-OMe), 3.73 (t, J = 5.4 Hz, 2H, H-8″), 2.54 (t, J = 7.2 Hz, 2H, H-2″), 1.76–1.66 (m, 8H, H-3″, 5″, 6″, 7″), 1.51–1.43 (m, 2H, H-4″). ^13^C-NMR (75.00 MHz, CDCl_3_) *δ*_ppm_:175.83 (C-4), 171.47 (C-1″), 159.73 (C-4′), 156.68 (C-9), 154.59 (C-7), 152.74 (C-2), 135.11 (4a, 4b, 4c), 133.59 (2a, 6a, 2b, 6b, 2c, 6c), 130.65 (3a, 5a, 3b, 5b, 3c, 5c), 130.14 (C-2′, 6′), 127.68 (C-5), 125.10 (C-1′), 122.87 (C-3), 122.19 (C-6), 119.58 (C-10), 118.91, 117.77 (1a, 1b, 1c), 114.07 (C-3′, 5′), 111.02 (C-8), 55.37 (-OCH_3_), 34.22 (C-2″), 30.31 (C-7″), 30.10 (C-4″), 28.61 (C-5″), 24.54 (C-3″), 22.65 (C-6″), 22.98 (C-8″).

7-O-(9-bromononanoyl)-formononetin-triphenylphosphine coupling(2d). Synthesized by 1d and triphenylphosphine. Yield:29.26%.

^1^H-NMR (300.00 MHz, CDCl_3_) *δ*_ppm_:8.30 (d, J = 9.0 Hz, 1H, H-5), 7.98 (s, 1H, H-2), 7.89–7.79 (m, 9H, H-3a, 4a, 5a, 3b, 4b, 5b, 3c, 4c, 5c), 7.72–7.66 (m, 6H, H-2a, 6a, 2b, 6b, 2c, 6c), 7.51–7.48 (m, 2H, H-2′, 6′), 7.28 (d, J = 2.1 Hz, 1H, H-8), 7.14 (dd, J = 8.7, 2.1 Hz, 1H, H-6), 6.99–6.96 (m, 2H, H-3′, 5′), 3.84 (s, 3H, 4′-OMe), 3.80 (t, J = 5.7 Hz, 2H, H-9″), 2.58 (t, J = 7.2 Hz, 2H, H-2″), 1.86–1.25 (m, 12H, H-3″, 4″, 5″, 6″, 7″, 8″). ^13^C-NMR (75.00 MHz, CDCl_3_) *δ*_ppm_:175.83 (C-4), 171.53 (C-1″), 159.75 (C-4′), 156.70 (C-9), 154.61 (C-7), 152.73 (C-2), 135.09 (4a, 4b, 4c), 133.62 (2a, 6a, 2b, 6b, 2c, 6c), 130.64 (3a, 5a, 3b, 5b, 3c, 5c), 130.15 (C-2′, 6′), 127.73 (C-5), 125.13 (C-1′), 123.89 (C-3), 122.22 (C-10), 119.57, 118.98 (1a, 1b, 1c), 117.84 (C-6), 114.08 (C-3′, 5′), 111.95 (C-8), 55.38 (-OCH_3_), 34.30 (C-2″), 30.47 (C-8″), 30.26 (C-6″), 28.85 (C-4″), 24.62 (C-5″), 23.05 (C-7″), 22.73 (C-9″).

7-O-(10-bromodecanoyl)-formononetin-triphenylphosphine coupling (2e) was synthesized using 1e and triphenylphosphine with a yield of 67.64%.

^1^H-NMR (300.00 MHz, CDCl_3_) *δ*_ppm_:8.29 (d, J = 8.7 Hz, 1H, H-5), 7.98 (s, 1H, H-2), 7.85–7.76 (m, 9H, H-3a, 4a, 5a, 3b, 4b, 5b, 3c, 4c, 5c), 7.72–7.66 (m, 6H, H-2a, 6a, 2b, 6b, 2c, 6c), 7.51–7.48 (m, 2H, H-2′, 6′), 7.28 (d, J = 2.1 Hz, 1H, H-8), 7.14 (dd, J = 8.7, 2.1 Hz, 1H, H-6), 6.99–6.95 (m, 2H, H-3′, 5′), 3.84 (s, 3H, 4′-OMe), 3.74 (t, J = 2.5 Hz, 2H, H-10″), 2.58 (t, J = 7.5 Hz, 2H, H-2″), 1.74–1.67 (m, 6H, H-3″, 8″, 9″), 1.26–1.25 (m, 8H, H-4″, 5″, 6″, 7″). ^13^C-NMR (75.00 MHz, CDCl_3_) *δ*_ppm_:175.89 (C-4), 171.59 (C-1″), 159.80 (C-4′), 156.75 (C-9), 154.66 (C-7), 152.77 (C-2), 135.13 (4a, 4b, 4c), 133.75 (2a, 6a, 2b, 6b, 2c, 6c), 130.67 (3a, 5a, 3b, 5b, 3c, 5c), 130.21 (C-2′, 6′), 127.79 (C-5), 125.19 (C-1′), 123.92 (C-3), 122.27 (C-6), 119.61 (C-10), 119.02, 117.88 (1a, 1b, 1c), 114.13 (C-3′, 5′), 111.04 (C-8), 55.41 (-OCH_3_), 34.39 (C-2″), 30.59 (C-5″), 30.38 (C-9″), 29.10C-7″/C-4″), 28.96 (C-6″), 24.75 (C-3″), 22.95 (C-10″), 22.72 (C-8″).

7-O-(11-bromoundecanoyl)-formononetin-triphenylphosphine coupling (2f) was synthesized using 1f and triphenylphosphine with a yield of 28.17%.

^1^H-NMR (300.00 MHz, CDCl_3_) *δ*_ppm_:8.28 (d, J = 8.79 Hz, 1H, H-5), 7.97 (s, 1H, H-2), 7.84–7.75 (m, 9H, H-3a, 4a, 5a, 3b, 4b, 5b, 3c, 4c, 5c), 7.71–7.65 (m, 6H, H-2a, 6a, 2b, 6b, 2c, 6c), 7.51–7.46 (m, 2H, H-2′, 6′), 7.27 (d, J = 2.4 Hz, 1H, H-8), 7.13 (dd, J = 8.7, 2.1 Hz, 1H, H-6), 6.99–6.94 (m, 2H, H-3′, 5′), 3.83 (s, 3H, 4-OMe), 3.74 (t, J = 2.9 Hz, 2H, H-11″), 2.58 (t, J = 7.5 Hz, 2H, H-2″)1.77–1.67 (m, 4H, H-3″, 10″)1.37–1.24 (m, 12H, H-4″, 5″, 6″, 7″, 8′, 9″). ^13^C-NMR (75.00 MHz, CDCl_3_) *δ*_ppm_:175.81 (C-4), 171.54 (C-1″), 159.71 (C-4′), 156.67 (C-9), 154.59 (C-7), 152.74 (C-2), 135.1 (C-4a, 4b, 4c), 133.69 (C-2a, 6a, 2b, 6b, 2c, 6c, )130.62 (C-3a, 5a, 3b, 5b, 3c, 5c), 130.13 (C-2′, 6′), 127.69 (C-5), 125.08 (C-1′), 123.84 (C-3), 122.18 (C-6), 119.54 (C-10), 118.91, 117.77 (C-1a, 1b, 1c), 114.04 (C-3′5′), 110.98 (C-8), 55.40 (-OCH_3_), 34.35 (C-2″), 30.56 (C-10″)30.35 (C-5″), 29.24 (C-8″), 29.15 (C-4″/7″), 28.96 (C-6″), 24.73 (C-3″), 22.65 (C-9″), 22.95 (C-11″).

7-O-(12-bromododecanoyl)-formononetin-triphenylphosphine coupling (2g) was synthesized using 1g and triphenylphosphine, with a yield of 28.42%.

^1^H-NMR (300.00 MHz, CDCl_3_) *δ*_ppm_:8.28 (d, J = 8.7 Hz, 1H, H-5), 7.97 (s, 1H, H-2), 7.85–7.74 (m, 9H, H-3a, 4a, 5a, 3b, 4b, 5b, 3c, 4c, 5c), 7.71–7.65 (m, 6H, H-1a, 6a, 1b, 6b, 1c, 6c), 7.50–7.46 (m, 2H, H-2′, 6′), 7.27 (d, J = 2.1 Hz, 1H, H-8), 7.13 (dd, J = 9.0, 2.4 Hz, 1H, H-6), 6.98–6.93 (m, 2H, H-3′, 5′), 3.83 (s, 3H, 4-OMe), 3.72 (t, J = 9.3 Hz, 2H, H-12″), 2.58 (t, J = 7.5 Hz, 2H, H-2″), 1.78–1.68 (m, 4H, H-3″, 11″), 1.38–1.21 (m, 14H, H-4″, 5″, 6″, 7″, 8″, 9″, 10″). ^13^C-NMR (75.00 MHz, CDCl_3_) *δ*_ppm_:175.72 (C-4), 171.47 (C-1″), 159.63 (C-4′), 156.59 (C-9), 154.52 (C-7), 152.68 (C-2), 135.05 (C-4a, 4b, 4c), 133.59 (C-2a, 6a, 2b, 6b, 2c, 6c, )130.58 (C-3a, 5a, 3b, 5b, 3c, 5c), 130.05 (C-2′, 6′), 127.59 (C-5), 124.98 (C-1′), 123.76 (C-3), 122.10 (C-6), 119.47 (C-10), 118.79, 117.65 (C-1a, 1b, 1c), 113.96 (C-3′, 5′), 110.91 (C-8), 55.33 (-OCH_3_), 34.29 (C-2″), 30.51 (C-11″), 30.30 (C-6″), 29.62 (C-5″), 29.23 (C-9″), 29.07 (C-4″/8″), 28.92 (C-7″), 24.67 (C-3″), 22.61 (C-10″), 22.24 (C-12″).

Then, 30 mg of formononetin was placed in a reaction vial with 15 mL of dichloromethane. According to the molar ratio (arnica: fatty acid: EDCI: DMAP = 1:1.5:4:2), the corresponding bromo-acid (n-hexanoic acid-lauric acid), EDCI, and DMAP were added to make it completely soluble in the dichloromethane and then reacted at room temperature for 1–4 h; TLC confirmed that the reaction was complete. The solvent was recovered under reduced pressure, and the clarified solution produced white crystals. Purification was performed by silica gel column chromatography (wet loading column, dry sampling separation conditions: petroleum ether (60–90 °C): acetone = 10:1). The collected eluate was subjected to TLC, and the solvent was recovered under reduced pressure to obtain fatty acids of different chain lengths (3a~3g).

7-O-hexanoyl-formononetin (3a) was synthesized from formononetin and hexanoic acid, with a yield of 84.42%.

^1^H-NMR (300.00 MHz, CDCl_3_) *δ*_ppm_:8.32 (d, J = 8.7 Hz, 1H, H-5), 7.98 (s, 1H, H-2), 7.53–7.48 (m, 2H, H-2′, 6′), 7.29 (d, J = 1.8 Hz, 1H, H-8), 7.15 (dd, J = 8.7, 2.1 Hz, 1H, H-6), 7.00–6.95 (m, 2H, H-3′, 5′), 3.84 (s, 3H, 4′-OMe), 2.61 (t, J = 7.5 Hz, 2H, H-2″), 1.84–1.74 (m, 2H, H-3″), 1.45–1.38 (m, 2H, H-4″, 5″), 0.97–0.92 (m, J = 9.00, 3H, 6″-H). ^13^C-NMR (75.00 MHz, CDCl_3_) *δ*_ppm_:176.31 (C-4), 172.00 (C-1″), 160.26 (C-4′), 157.20 (C-9), 155.10 (C-7), 153.10 (C-2), 130.65 (C-2′, 6′), 128.34 (C-5), 125.69 (C-1′), 124.38 (C-3), 122.77 (C-6), 120.01 (C-10), 114.57 (C-3′, 5′), 111.39 (C-8), 55.90 (-OCH_3_), 34.90 (C-2″), 31.76 (C-4″), 25.02 (C-3″), 22.84 (C-5″), 14.45 (C-6″).

7-O-pivaloyl-formononetin (3b) was synthesized from formononetin and n-heptanoic acid, with a yield of 86.54%.

^1^H-NMR (300.00 MHz, CDCl_3_) *δ*_ppm_:8.32 (d, J = 8.7 Hz, 1H, H-5), 7.98 (s, 1H, H-2), 7.53–7.48 (m, 2H, H-2′, 6′), 7.29 (d, J = 2.1 Hz, 1H, H-8), 7.15 (dd, J = 8.7, 2.1 Hz, 1H, H-6), 7.00–6.95 (m, 2H, H-3′, 5′), 3.85 (s, 3H, 4′-OMe), 2.61 (t, J = 7.5 Hz, 2H, H-2″), 1.83–1.73 (m, 2H, H-3″)1.48–1.39 (m, 2H, H-4″), 1.37–1.32 (m, 2H, H-5″, 6″), 0.94–0.90 (m, 3H, H-7″). ^13^C-NMR (75.00 MHz, CDCl_3_) *δ*_ppm_:175.89 (C-4), 171.58 (C-1″), 159.83 (C-4′), 156.78 (C-9), 154.68 (C-7), 152.71 (C-2), 130.23 (C-2′, 6′), 127.92 (C-5), 125.27 (C-1′), 123.95 (C-3), 122.34 (C-6), 119.58 (C-10), 114.14 (C-3′, 5′), 111.00 (C-8), 55.43 (-OCH_3_), 34.51 (C-2″), 31.53 (C-5″), 28.85 (C-4″), 24.87 (C-3″), 22.59 (C-6″), 14.13 (C-7″).

7-O-octanoyl-formononetin (3c) was synthesized from formononetin and n-octanoic acid, with a yield of 86.92%.

^1^H-NMR (300.00 MHz, CDCl_3_) *δ*_ppm_:8.32 (d, J = 8.7 Hz, 1H, H-5), 7.97 (s, 1H, H-2), 7.52–7.47 (m, 2H, H-2′, 6′), 7.29 (d, J = 2.1 Hz, 1H, H-8), 7.15 (dd, J = 8.7, 2.1 Hz, 1H, H-6), 6.99–6.95 (m, 2H, H-3′, 5′), 3.85 (s, 3H, 4′-OMe), 2.61 (t, J = 7.5 Hz, 2H, H-2″), 1.83–1.73 (m, 2H, H-3″), 1.43–1.32 (m, 2H, H-4″, 5″, 6″, 7″), 0.93–0.88 (m, 3H, H-8″). ^13^C-NMR (75.00 MHz, CDCl_3_) *δ*_ppm_:176.33 (C-4), 172.02 (C-1″), 160.26 (C-4′), 157.21 (C-9), 155.10 (C-7), 153.15 (C-2), 130.66 (C-2′, 6′), 128.36 (C-5), 125.69 (C-1′), 124.38 (C-3), 122.77 (C-6), 120.02 (C-10), 114.57 (C-3′, 5′), 111.43 (C-8), 55.86 (-OCH_3_), 34.94 (C-2″), 34.18 (C-6″), 29.58 (C-4″), 29.45 (C-5″), 25.34 (C-3″), 23.14 (C-7″), 14.61 (C-8″).

7-O-nonanoyl-formononetin (3d) was synthesized from formononetin and n-onanoic acid, with a yield of 86.90%.

^1^H-NMR (300.00 MHz, CDCl_3_) *δ*_ppm_:8.32 (d, J = 8.7 Hz, 1H, H-5), 7.98 (s, 1H, H-2), 7.53–7.48 (m, 2H, H-2′, 6′), 7.29 (d, J = 2.1 Hz, 1H, H-8), 7.15 (dd, J = 8.7, 2.1 Hz, 1H, H-6), 7.00–6.95 (m, 2H, H-3′, 5′), 3.85 (s, 3H, 4′-OMe), 2.61 (t, J = 7.5 Hz, 2H, H-2″), 1.83–1.73 (m, 2H, H-3″), 1.40–1.29 (m, 2H, H-4″, 5″, 6″, 7″, 8″), 0.92–0.87 (m, 3H, H-9″). ^13^C-NMR (75.00 MHz, CDCl_3_) *δ*_ppm_:175.91 (C-4), 171.59 (C-1″), 159.84 (C-4′), 156.79 (C-9), 154.69 (C-7), 152.73 (C-2), 130.24 (C-2′, 6′), 127.24 (C-5), 125.28 (C-1′), 123.96 (C-3), 122.35 (C-6), 119.60 (C-10), 114.15 (C-3′, 5′), 110.97 (C-8), 55.48 (-OCH_3_), 34.52 (C-2″), 31.92 (C-7″), 29.32 (C-5″), 29.23 (C-6″), 29.20 (C-4″), 24.92 (C-3″), 22.77 (C-8″), 14.22 (C-9″).

7-O-n-decanoyl-formononetin (3e) was synthesized from formononetin and n-decanoic acid, with a yield of 93.90%.

^1^H-NMR (300.00 MHz, CDCl_3_) *δ*_ppm_:8.32 (d, J = 9.0 Hz, 1H, H-5), 7.98 (s, 1H, H-2), 7.52–7.48 (m, 2H, H-2′, 6′), 7.29 (d, J = 2.1 Hz, 1H, H-8), 7.15 (dd, J = 8.7, 2.1 Hz, 1H, H-6), 7.00–6.95 (m, 2H, H-3′, 5′), 3.85 (s, 3H, 4′-OMe), 2.61 (t, J = 7.5 Hz, 2H, H-2″), 1.83–1.73 (m, 2H, H-3″), 1.43–1.28 (m, 2H, H-4″, 5″, 6″, 7″, 8″, 9″), 0.91–0.87 (m, 3H, H-10″).^13^C-NMR (75.00 MHz, CDCl_3_) *δ*_ppm_:175.89 (C-4), 171.58 (C-1″), 159.84 (C-4′), 156.78 (C-9), 154.69 (C-7), 152.67 (C-2), 130.24 (C-2′, 6′), 127.93 (C-5), 125.27 (C-1′), 123.96 (C-3), 122.35 (C-6), 119.59 (C-10), 114.15 (C-3′, 5′), 111.01 (C-8), 55.43 (-OCH_3_), 34.52 (C-2″), 31.98 (C-8″), 29.52 (C-5″), 29.37 (C-6″/7″), 29.19 (C-4″), 24.92 (C-3″), 22.79 (C-9″), 14.22 (C-10″).

7-O-undecanoyl-formononetin (3f) was synthesized from formononetin and n-undecanoic acid, with a yield of 96.07%.

^1^H-NMR (300.00 MHz, CDCl_3_) *δ*_ppm_:8.32 (d, J = 8.7 Hz, 1H, H-5), 7.98 (s, 1H, H-2), 7.53–7.48 (m, 2H, H-2′, 6′), 7.29 (d, J = 2.1 Hz, 1H, H-8), 7.15 (dd, J = 8.7, 2.1 Hz, 1H, H-6), 7.00–6.95 (m, 2H, H-3′, 5′), 3.85 (s, 3H, 4′-OMe), 2.61 (t, J = 7.5 Hz, 2H, H-2″), 1.83–1.73 (m, 2H, H-3″), 1.43–1.25 (m, 2H, H-4″, 5″, 6″, 7″, 8″, 9″, 10″), 0.91–0.86 (m, 3H, H-11″). ^13^C-NMR (75.00 MHz, CDCl_3_) *δ*_ppm_:175.83 (C-4), 171.53 (C-1″), 159.80 (C-4′), 156.74 (C-9), 154.65 (C-7), 152.69 (C-2), 130.19 (C-2′, 6′), 127.88 (C-5), 125.22 (C-1′), 123.93 (C-3), 122.31 (C-6), 119.55 (C-10), 114.11 (C-3′, 5′), 110.97 (C-8), 55.43 (-OCH_3_), 34.48 (C-2″), 31.99 (C-9″), 29.64 (C-5″), 29.54 (C-7″), 29.40 (C-6″), 29.33 (C-8″), 29.17 (C-4″), 24.98 (C-3″), 22.78 (C-10″), 14.21 (C-11″).

7-O-dodecanoyl-formononetin (3g) was synthesized from formononetin and n-lauric acid, with a yield of 88.71%.

^1^H-NMR (300.00 MHz, CDCl_3_) *δ*_ppm_:8.32 (d, J = 8.7 Hz, 1H, H-5), 7.98 (s, 1H, H-2), 7.52–7.48 (m, 2H, H-2′, 6′), 7.29 (d, J = 2.1 Hz, 1H, H-8), 7.15 (dd, J = 8.7, 2.1 Hz, 1H, H-6), 7.00–6.95 (m, 2H, H-3′, 5′), 3.85 (s, 3H, 4′-OMe), 2.61 (t, J = 7.5 Hz, 2H, H-2″), 1.83–1.73 (m, 2H, H-3″), 1.42–1.26 (m, 2H, H-4″, 5″, 6″, 7″, 8″, 9″, 10″, 11″), 0.90–0.86 (m, 3H, H-12″). ^13^C-NMR (75.00 MHz, CDCl_3_) *δ*_ppm_:175.94 (C-4), 171.60 (C-1″), 159.86 (C-4′), 156.81 (C-9), 154.71 (C-7), 152.75 (C-2), 130.25 (C-2′, 6′), 127.96 (C-5), 125.30 (C-1′), 123.96 (C-3), 122.36 (C-6), 119.61 (C-10), 114.17 (C-3′, 5′), 110.97 (C-8), 55.49 (-OCH_3_), 34.54 (C-2″), 32.04 (C-10″), 29.73 (C-7″/8″), 29.57 (C-6″), 29.47 (C-5″), 29.37 (C-9″), 29.21 (C-4″), 24.93 (C-3″), 22.82 (C-11″), 14.24 (C-12″).

### 4.3. Cell Lines

The human embryonic kidney cells (HEK-293), human non-small-cell lung cancer (A549), human breast cancer cells (MCF-7), and human prostate cancer (PC-3M) cells used in this study were obtained from the Beijing BNCC Biotechnology Research Institute (Beijing, China), and human gastric cancer cells (HGC-27) were obtained from Dalian Meilun Biotechnology Co., Ltd. (Dalian, China).

### 4.4. Cell Viability Assay

The cell lines used in this study (A549, MCF-7, PC-3M, HGC-27, HEK-293) were removed from the CO_2_ incubator and seeded into 96-well plates at a density of 5 × 10^4^/mL. Following a 24-h incubation period under standard CO_2_ conditions, we added 100 μL/mL of sample solutions at various concentrations (3.125, 6.26, 12.5, 25, 50, 100 μmol/L) to each well. Each concentration was tested in six replicates. The control group received 0.1% DMSO in serum-free medium (SFM). After 48 h of the drug effect, 10 µL of MTT solution was added to each well under light-avoidance conditions, and incubation was continued under light-avoidance conditions for 4 h; the supernatant was discarded, and 150 μL of DMSO was added to dissolve the resulting formazan crystals. After 10 min of shaking to ensure the complete dissolution and uniform color development of purple crystals, the absorbance (OD) was measured at 490 nm using a microplate reader.

The inhibition rate (%) was calculated using the following formula:

The inhibition rate was calculated as: Inhibition rate (%) = [(OD value of control group-OD value of administered group)/(OD value of control group-OD value of zeroing group)] × 100%, where OD value of zeroing group represents the zeroing group.

The IC_50_ values of the drugs were calculated by fitting using GraphPad Prism v.6.01 (GraphPad Software, San Diego, CA, USA), and it was used as a criterion for selecting the appropriate drug concentration for subsequent experiments.

### 4.5. Network Pharmacology

MTT assay results demonstrated that all synthesized derivatives exhibited significant inhibitory activity on lung cancer cells. Consequently, lung cancer was selected as the model for mitochondrial gene screening in this study.

#### 4.5.1. Tumor Cell and Mitochondrial Crossover Gene Screening

Gene expression data from lung adenocarcinoma patients and healthy individuals were obtained from the Cancer Genome Atlas (TCGA) database (https://www.cancer.gov/ccg/research/genome-sequencing/tcga (accessed on 22 January 2025)). Following data preprocessing, including removal of non-clinical information and duplicate entries, differential expression analysis was conducted. A total of 1136 human mitochondrial genes were retrieved from the Mito Carta 3.0 database [49] and cross-referenced with the TCGA dataset to identify genes associated with both lung cancer and mitochondrial function. Differentially expressed mitochondrial genes specific to lung cancer were screened using Venny 2.1 (https://bioinfogp.cnb.csic.es/tools/venny/ (accessed on 22 January 2025)).

#### 4.5.2. GO and KEGG Pathway Enrichment Analysis of Overlapping Genes

Gene ontology (GO) functional enrichment analysis was performed to elucidate the biological roles of the overlapping genes, categorized into biological processes (BPs), cellular components (CCs), and molecular function (MF). Additionally, Kyoto Encyclopedia of Genes and Genomes (KEGG) pathway enrichment analysis was conducted to identify the major signaling pathways influenced by those overlapping mitochondrial and tumor-related genes.

#### 4.5.3. Core Gene Screening and Protein–Protein Interaction (PPI) Network Construction

Protein–protein interaction (PPI) analysis of overlapping genes was conducted using the STRING database (https://cn.string-db.org/). Core genes were identified by integrating three analytical algorithms—CytoNCA, Mcode, and Cytohubba—within the Cytoscape software version 3.10.0 platform. The identified core genes were then imported into the STRING database to construct the PPI network. Among these, the gene with the highest degree centrality was selected as the key protein target for molecular docking with the synthesized derivatives [50].

### 4.6. Molecular Docking

Following evaluation using web-based pharmacological tools, core targets with high relevance scores were selected for molecular docking analysis.

The crystal structure of the *SHMT2* protein (PDB ID:8GKT) was retrieved from the Protein Data Bank database (https://www.rcsb.org/ (accessed on 10 February 2025)) [51]. PyMOL software version 3.1.0 was used to process the target protein structure, stripping the embedded small molecules of the target protein as well as removing the crystalline water molecules and a series of other treatments, which were saved as an 8GKT.pdb file; the derivatives were used to draw the ligand small molecules with a three-dimensional structure using ChemDraw 21.0.0 (ChemDraw Software, PerkinElmer, Waltham, MA, USA); and the target protein receptor was hydrogenated and charged by running Vina 1.1.2 (AutoDock Vina Software, Scripps Research, San Diego, CA, USA) software to generate the 8GKT.pdbqt file. Ligand molecules were constructed in a three-dimensional form using ChemDraw 21.0.0 (PerkinElmer, Waltham, MA, USA). Both the receptor protein and ligand molecules were prepared for docking through hydrogenation and charge assignment using AutoDock Vina 1.1.2 (Scripps Research, San Diego, CA, USA), resulting in pdbqt files.

The ligand molecules were subjected to energy optimization, including hydrogenation and charge assignment, to minimize their energy and generate a pdbqt file. The conformation with the lowest binding free energy was selected to analyze the interactions between the macromolecule and the target proteins [52].

The docking simulation was carried out using AutoDock Vina 1.1.2 under a 3D semi-flexible model, where the receptor molecule was considered rigid and the ligand flexible. The dimensions of the Gridbox were set to 93.0 × 80.25 × 79.5 (Å). The number of docking runs was adjusted to 20, while all other parameters were maintained at their default settings [53]. The docking pocket centers for each protein receptor were defined as follows:for the small molecule, X:97.189, Y:0.416, Z:43.436; for the protein, X:−16.847, Y:71.004, Z:2.697). The most stable docking conformation with the lowest binding energy was selected for each compound, yielding data such as binding energy between the compounds and the target receptor. Subsequently, docking results were imported into PyMOL software to visualize and generate protein–ligand complexes. Protein–ligand interaction data were version 3.1.0 analyzed using the Protein–Ligand Interaction Profiler (PLIP) platform, exported in .pse format, and visualized using PyMOL software version 3.1.0.

### 4.7. Molecular Dynamics Simulations

In this study, molecular dynamics (MD) simulations were performed using GROMACS 2022. Force field parameters were generated via the pdb2gmx tool in GROMACS 2022 and the AutoFF web server. During the simulation, the molecular parameters of the receptor protein were assigned using the CHARMM36 force field [54], while the ligand parameters were generated using the CGenff force field. The system was solvated in a TIP3P cubic water box with a 1 nm buffer around the solute [55]. Ions were added using the gmx genion tool to neutralize the system’s net charge. Long-range electrostatic interactions were handled using the Particle Mesh Ewald (PME) method with the cutoff distance set to 1 nm. All bond constraints were applied using the SHAKE algorithm, and the integration time step was set to 1 fs with the Verlet leapfrog algorithm. Before the production MD simulation, the system underwent energy minimization, consisting of 3000 steps of steepest descent followed by 2000 steps of conjugate gradient optimization. The minimization procedure was carried out in stages:first, the solute was restrained while water molecules were minimized; next, counterions were restrained during minimization; finally, full-system minimization was performed without restraints. The MD simulation was conducted under NPT ensemble conditions at a temperature of 310 K and a constant pressure for a total simulation time of 50 ns. During the simulation, various structural and dynamic properties were analyzed using the following Gromacs tools:g-rmsd for root mean square deviation (RMSD), g-rmsf for root square fluctuation (RMSF), and g-hbond for solvent-accessible surface area (SASA). The binding free energy after system equilibration was calculated using the MM/PBSA method. The MM/PBSA binding free energy of the compound was computed using the g_mmpbsa package within Gromacs.

## 5. Conclusions

The principal innovation of this study is the successful design and synthesis of 21 novel formononetin derivatives, whose chemical structures were unequivocally characterized by comprehensive ^1^H-NMR and ^13^C-NMR spectroscopic analysis. The cytotoxic potential of these compounds was systematically evaluated through MTT viability assays. Network pharmacological analysis was employed to identify core target genes, among which *SHMT2* was recognized as a critical target for tumor treatment. Through integrated network pharmacology approaches, serine hydroxymethyltransferase 2 (*SHMT2*) was identified as a pivotal molecular target implicated in tumor progression. Molecular docking simulations demonstrated high-affinity binding interactions between the optimized lead compound and the *SHMT2* active site, as evidenced by favorable binding energies and stable molecular interactions. The stability and structural integrity of the derivative-protein complex were confirmed through molecular dynamics simulations conducted over a 100 ns period. These computational findings strongly suggest that the final compound 2c represents a promising therapeutic candidate for targeted lung cancer treatment. In the present study, initial screening was performed by using a panel of five cell lines to assess broad-spectrum activity. However, the generalizability of these findings to other cancer types or primary tumors requires additional validation. Furthermore, comprehensive investigations remain necessary to thoroughly evaluate the anticancer efficacy of compound 2c against established lung cancer cell lines.

## Figures and Tables

**Table 1:** Cytotoxic activity of derivative intermediates and end products (half inhibitory concentration, μM).

Number	Cell Name				
	HGC-27	MCF-7	A549	PC-3M	HEK-293
1a	83.34 ± 8.32	-	-	-	83.43 ± 8.61
1b	79.14 ± 5.65	-	-	-	70.72 ± 5.61
1c	31.43 ± 4.93	-	>100	-	67.96 ± 5.33
1d	79.25 ± 9.27	-	86.20 ± 6.10	-	74.38 ± 4.28
1e	66.06 ± 7.33	-	65.37 ± 5.01	83.99 ± 11.0	95.07 ± 7.97
1f	55.58 ± 2.43	-	74.59 ± 11.44	-	95.72 ± 8.09
1g	95.45 ± 6.23	-	53.57 ± 5.15	>100	59.94 ± 7.79
2a	13.39 ± 2.77	72.83 ± 6.25	28.79 ± 2.45	55.16 ± 4.40	44.82 ± 2.39
2b	32.12 ± 5.96	46.49 ± 4.94	42.22 ± 3.69	69.18 ± 3.98	46.22 ± 3.99
2c	18.62 ± 1.60	42.61 ± 9.44	12.19 ± 1.52	21.73 ± 1.25	76.99 ± 8.26
2d	29.14 ± 2.58	56.75 ± 4.04	31.21 ± 4.40	66.99 ± 6.97	46.99 ± 8.41
2e	39.16 ± 2.68	85.51 ± 4.99	38.96 ± 1.65	97.94 ± 6.33	64.05 ± 3.47
2f	30.43 ± 2.12	52.28 ± 6.85	20.61 ± 1.21	27.86 ± 2.69	51.69 ± 5.48
2g	29.36 ± 2.52	46.58 ± 4.95	23.64 ± 2.25	41.16 ± 4.40	59.57 ± 5.65
3a	49.50 ± 3.35	-	-	61.97 ± 8.71	-
3b	65.36 ± 3.88	-	96.44 ± 7.37	-	-
3c	59.98 ± 7.82	-	82.96 ± 5.89	-	-
3d	80.22 ± 4.80	-	78.08 ± 6.21	58.34 ± 5.36	78.28 ± 9.74
3e	48.59 ± 5.30	-	74.02 ± 11.92	-	-
3f	45.39 ± 5.24	84.99 ± 5.74	70.13 ± 9.67	46.71 ± 9.24	74.96 ± 5.49
3g	-	67.75 ± 8.92	66.94 ± 9.70	50.35 ± 5.78	50.35 ± 6.13
FMN	29.55 ± 1.17	-	83.02 ± 6.25	76.39 ± 6.47	39.60 ± 3.26
DOX	5.97 ± 0.43	13.21 ± 0.43	15.41 ± 1.06	0.168 ± 0.06	-
5-FU	36.24 ± 3.02	-	22.92 ± 3.54	48.05 ± 3.76	>100

**Table 2:** Results of the interaction of each derivative with the receptor target protein 8GKT.

NO.	Name	Synergy	Hydrophobicity
2a	7-O-(6-Bromohexanoyl)-formononetin sapogenins-triphenylphosphine couples	−9.3 Kcal/mol	ASN-408, ALA-227, ALA-418, TYR-106, TYR-105, PRO-321, PRO-167, LEU-172, LEU-166, ILE-183
2b	7-O-(7-Bromoheptanoyl)-formononetin sapogenins-triphenylphosphine couples	−8.6 Kcal/mol	LYS-409, TYR-105, PRO-421, ALA-418, LYS-181, PHE-317, LEU-172, LEU-166, ILE-183, TYR-176
2c	7-O-(8-Bromooctanoyl)-formononetin sapogenins-triphenylphosphine couples	−9.4 Kcal/mol	PHE-320, PHE-317, ILE-183, LEU-166, LEU-172, ALA-227, ASN-408, GLU-98, TYR-96, TYR-105
2d	7-O-(9-Bromononanoyl)-formononetin sapogenins-triphenylphosphine couples	−9.2 Kcal/mol	LEU-166, TYR-106, ALA-418, PHE-320, ASN-408, TYR-105
2e	7-O-(10-Bromodecanoyl)-formononetin sapogenins-triphenylphosphine couples	−9.0 Kcal/mol	LEU-172, PHE-320, LEU-166, PHE-317, ILE-183, ASP-313, TYR-105, LYS-103
2f	7-O-(11-Bromoundecanoyl)-formononetin sapogenins-triphenylphosphine couples	−9.1 Kcal/mol	ALA-418, PHE-320, ASN-408, TYR-105, LYS-409, PRO-167, LEU-166, ALA-227
2g	7-O-(12-Bromododecanoyl)-formononetin sapogenins-triphenylphosphine couplings	−8.9 Kcal/mol	TYR-100, LYS-103, PHE-317, LEU-172, PHE-320, PRO-321, ILE-183, LEU-166, TYR-105
FMN	formononetin	−7.9 Kcal/mol	TYR-106, TYR-105, PHE-320, PRO-321, LEU-172, LEU-166

## Data Availability

The data that support the findings of this study are available from the corresponding author upon reasonable request.
